# The impact of nursing intervention on the care burden experienced by mothers of children with epilepsy: a family-centered empowerment model

**DOI:** 10.1186/s12912-025-03532-9

**Published:** 2025-07-09

**Authors:** Sahar Mahmoud Elkhedr Abdelgawad, Samar Abd-Elrahman Radwan, Zeinab El-Sayed Hafez Elsayed

**Affiliations:** Pediatric Nursing, Faculty of Nursing, Tanta University, Tanta, Egypt

**Keywords:** Care burden, Children, Family centered empowerment model, Epilepsy, Mothers

## Abstract

**Background:**

Family-Centered Empowerment Model (FCEM) is a model which promotes knowledge, and skills which help family members to gain better understanding of the child’s health problems that is consequently reduce care burden level.

**Aim:**

The current study’ aim was to evaluate the impact of nursing interventions on the care burden experienced by mothers of children with epilepsy: A family Centered empowerment model.

**Design:**

A quasi experimental design was utilized.

**Subjects:**

A purposive sample of 60 mothers accompanied their children with epilepsy was involved.

**Setting:**

This study was carried out at Neurological Unit, Pediatric Medical Department & Neurological Outpatient Clinic of Tanta Main University Hospital.

**Tools:**

**Tool I** Epilepsy Knowledge Questionnaire.

**Tool II:**

Mothers’ Reported Practice Regarding Epilepsy.

**Tool III:**

Zarit Burden Short Version Interview.

**Results:**

After implementing FCEM all of the mothers’ showed significant improvement in their knowledge levels, both immediately posttest and at three months follow-up compared to about one third of them who showed adequate knowledge at pre-test. Most of mothers had unsatisfactory practice before implementing FCEM. However, these practice had improved notably for the majority both immediately and after three months post implementing FCEM. There was a significant decrease in mothers’ care burden following implementation of FCEM as compared to pre- implementing FCEM.

**Conclusion:**

Mothers knowledge and reported practice were improved after implementing FCCM. Implementing family-centered empowerment model had significantly decreased mothers’ care burden of their children with epilepsy.

**Recommendation:**

Incorporate family-centered empowerment model into routine nursing care for children with epilepsy.

## Introduction

Epilepsy is one of brain disorder that characterized by frequent, spontaneous seizures brought on by heighted electrical activity in the brain. It occurs when the child has 2 or more seizures with unknown cause [1]. Because of its diverse genesis, neurological manifestations, and pathophysiology, epilepsy is not a single illness [2]. It causes repeated seizures, which are sudden and transient surges of abnormal excessive neuronal activity in the brain. They lead to clinical alteration of neurologic function [3]. 

Epilepsy is the commonest neurological disorder affecting 11 million children worldwide. One out of every150 children has epilepsy throughout their first 10 years of their life. Among low to middle income countries, the incidence is 149/100.000, while it is 43.4/ 100.00 in developed countries [4]. Prevalence of childhood epilepsy in Egypt was 123 /1000 per year with higher prevalence among children less than 12 years than adolescents [5]. Idiopathic epilepsy was found in more than half of the affected children (59.4%) [6].

Epilepsy causes in children vary. It was due to unknown cause in (67.6%), congenital (20%), trauma-related (4.7%), infection-related (4%), stroke and tumors (1.5%), and degenerative (0.7%) are the possible causes [7]. Depending on seizure type, epileptic children can exhibit a wide range of symptoms, including stiffness of the body, jerking motions of the arms and legs, and staring [8]. Management of epilepsy requires integration and cooperation among health care team and children’s care givers/ parents to improve child’s prognosis [9]. 

Parents especially mothers are mainly their children primary caregivers [10]. Stigma associated with disclosing epilepsy, as well, deficiency of reliable information regarding coping mechanisms, and the requirement for emotional and psychosocial support are examples of the challenges that epileptic children often experience [11]. In addition, caring for epileptic children is linked with severe burden and psychological problems which are extremely difficult and stressful experience among parents of epileptic children [12]. 

Care burden is another result of looking after children with epilepsy which may be physically, psychologically, or socially [13]. Care burden is a multi-factorial construct as it may be objective or subjective. Subjective burden includes the physical, mental, social, and emotional strains caregivers endure while carrying out their caregiving responsibilities, whereas objective burden refers to the time and money needed to provide care [14]. 

Shifting in the epilepsy care from traditional approaches to empowering children and their families, and promoting their involvement in the care process significantly improve children’s health [15]. Family-centered empowerment model is an Iranian health model that improve chronic diseases management [16]. This model is designed based on the effectiveness of the caregivers’ role based on the three characteristics which are motivational, self-problem as knowledge, attitude, and threats perception, and psychological as self-efficacy and self-esteem. It aims to empower all family members. It also, seeks to promote health of both family and epileptic children [17]. 

Pediatric nurses play an important role in supporting mothers of epileptic children by providing both medical and emotional assistance. They help reduce the care burden by educating mothers about seizure management, medication administration, and recognizing warning signs. During regular follow-ups, they ensure treatment compliance and offer reassurance during stressful times [18]. Pediatric nurses also, connect mothers with support groups and community resources, fostering a sense of shared experience and reducing isolation. By empowering mothers with knowledge, skills, and emotional support, pediatric nurses significantly improve care burden experienced by both children and their mothers [19]. 

### Significance of the study

Caring for children suffering from epilepsy is results in severe care burden among parents [20]. Family members especially mothers have many responsibilities in caring for children with epilepsy [21]. Mothers of epileptic children must work harder to find adequate and appropriate support for their children that may result in anxiety, frustration, or depression that increased burden level among those mothers [22]. Empowerment is important in helping mothers to manage stressful situations, deliver good care, and accomplish caring duties as effectively as possible while experiencing less psychological stress, exhaustion, and tension [16, 23]. In Egypt, limited attention is directed to empowering mothers to care for their epileptic children and there are no documented findings about this [24, 25]. Mothers’ level of education may have an impact on their ability to access, understand, appraise, remember, and apply information about Children’s health. In addition, clinical characteristics of the child’s condition may have an impact on the parents’ need for healthy life [26]. Hence the current study aimed to implement FCEM to lessen care burden of mothers of epileptic children.

### The study aim was

Evaluate the impact of nursing interventions using family centered empowerment model on the care burden experienced by mothers of children suffering from epilepsy.

### Research hypothesis

- Mothers’ knowledge is expected to be improved after implementing FCE.
- Mothers’ reported practice for their epileptic children is expected to be enhanced post implantation of FCEM.
- Implementing family centered care model is expected to decrease mothers’ care burden.

### Subject and methods

#### Research design

A quasi-experimental was utilized. It is a research design used to evaluate the effects of an intervention when random assignment to groups is not possible. In the current study, it was used to assess the effectiveness of FCEM on mothers’ knowledge, practice and care burden before and after intervention, “pretest, immediately posttest & three months posttest evaluation was used”.

#### Setting

The current study was implemented at Neurological Unit, Pediatric Medical Department & Neurological Outpatient Clinic of Tanta Main University Hospital, which is affiliated to the Ministry of Higher Education and Scientific Research.

#### Subjects

Purposive sampling of 60 mothers accompanied their epileptic children were included. Epileptic children’s total number is about 300 / year. The sample size and power analysis were calculated using Epi-info software statistical package, the level of confidence was determined at 90% with margin error of 10% and population proportion 50%.

### Inclusion criteria for children

- Age of children is between 4 and 8 years.
- Diagnosed & treated from epilepsy at least six months before conducting this study.

### Exclusion criteria for mothers

- Mothers who had any chronic diseases that may affect their care burden. “ex. Diabetes, Hypertension, kidney diseases etc.”

## Tools of data collection

### Three tools were used in the present study

**Tool [I]: Epilepsy Knowledge Questionnaire**: It included two sections:

**Part (1): Mothers & Children Socio-demographic characteristics**:

A. **For mothers**: age, level of educational, residence, job, marital status, consanguinity, income, number of family members.
B. **For children**: number of siblings, birth order, age, and sex.

**Part (2): Mothers’ knowledge about epilepsy and epileptic seizures**:

The researcher designed it after reviewing the related literatures. [2, 19, 25, 26] It included the following sub-parts:

A. **Knowledge regarding child’s history of epilepsy**: such as causes of epilepsy, child’s age at first seizure attack, timing, duration, and frequency of epileptic seizure. It also, included seizure triggering factors, health problems associated with epilepsy, history of hospitalization, type of seizure, and presence of aura.
B. **Knowledge about epilepsy and epileptic seizures**: such as meaning, causes, manifestations, complications, and treatment epilepsy. It also, included knowledge about triggers, signs and symptoms of aura, and safety measures during seizure.

The questionnaire sheet consisted of 10 questions; the score of every question was (0–2 grades). Score of correct and complete answer was (2), correct and incomplete answers’ score was (1), and wrong or not known answers’ score was (0). The total sum was (20). The cut-off point for the questionnaire was determined based on a percentage scoring method, commonly used in similar studies.

**Mothers ’ total knowledge score was calculated and classified as follow**:

- High knowledge level was considered from 75% and more. (15–20)
- Moderate knowledge level was considered from 50 to < 75%. (10-<15)
- Low knowledge level was considered < 50%. (0-<10)

**Tool [II]: Mothers’ Reported Practice Regarding Epilepsy**:

The researcher developed it after reviewing of recent and related literatures [20, 27, 28] to evaluate mothers’ reported practice for their epileptic children before, immediately and three months post implementing FCEM as follow:

- **Safety precautions before seizures including** notifying school or nursery about child illness, avoiding putting the child alone in a bathtub full of water, and keeping the child away from sharp objects.
- **Safety precautions during aura**: staying with the child until seizure attack ends, keeping harmful things away, and preventing child from leaving home.
- **Mothers’ practice during seizure as** pressing child tongue with tongue depressor, protecting child’s head from any injury, and loosing child’s tight clothes.
- **Practice after seizure such as** putting child in side lying position, checking child breathing, and reassuring child.

**Mothers’ reported practice scoring system was**:

- Correct and complete reported practice was scored (1).
- Incorrect or not done reported practice was scored (0).

**Mothers’ reported practice total score was calculated as follow**:

- Unsatisfactory practice < 75%.
- Satisfactory practice ≥ 75%.

**Tool [III]: The Zarit Burden Short Version Interview**:

It was developed by Bedard et al., (2011) [29], adapted and translated into Arabic by the researchers. The content validity index (CVI) was used to evaluate validity of the Arabic translated tool. Cronbach alpha test-retest was calculated, it was 0.941 that indicated high reliability of the translated tools.

It was used to assess the level of mothers’ care burden before, immediately and three months after implementing FCEM. It is a self-reported questionnaire which includes 22 items on a 5-point scale in which **Never was scored 0**,** Rarely was scored 1**,** Sometimes was scored 2**,** Quite Frequently was scored 3 & Nearly Always was scored 4.**

### Total score of mothers’ burden was classified as follow

- Little or no care burden was ranged between 0 and 21.
- Mild care burden ranged between 22 and 40.
- Moderate care burden ranged between 41 and 60.
- Severe care burden ranged between 61 and 88.

### Method

The study was completed using these steps.

1. **Administrative process**:

An official permission for collecting data was acquired from the responsible authorities before carrying out the current study.

2. **Ethical considerations**:

Ethical approval was acquired from the Faculty of Nursing Scientific Research Ethical Committee (code 234/4/2023). The study didn’t’ cause any harm for the participants. Mothers of children with epilepsy gave their informed consent. Confidentiality of information was maintained.

3. **Content validity & Reliability**:

Tools of the study were tested for clarity, and content validity by a jury of five experts in the field of the study. Pilot study was applied on 10% of the study sample. **Reliability of tools**: Cronbach’s alpha was utilized to check tools reliability which is 0.765 for knowledge, 0.844 for practice, 0.941 for care burden that indicated high reliability of the tools.

4. Data was gathered over a period of one year starting from July 2023 to July 2024.

### Study phases

It was conducted through the following four phases:

1. **Assessment Phase**: The researcher gather baseline information about mothers and their children with epilepsy using the study tools.
2. **Planning Phase**: This researcher set objectives of each session, preparing the environment and schedule meeting times. Prepare of the educational materials (lectures, printed booklet with illustrated pictures, videos, power point) & prepare the content of the study.
3. **Implementation Phase**:

- Family Centered Empowerment Model (FCEM) was implemented for mothers through holding successive sessions. The studied mothers were interviewed individually to collect baseline data. They were divided into 10 groups with 6 mothers in each group.
- Family Centered Empowerment Model was carried out through four sessions 2 sessions / week. Each session took between 30 and 45 min including discussion. Each session began with a review of the prior content and finished by a conclusion about the covered topics.

### The educational sessions were implemented as follow

#### The first session

A theoretical session that focused on providing mothers with knowledge about epilepsy meaning, causes, manifestations, and complications. It also included knowledge about epileptic seizures as timing, triggers, duration, manifestations of aura. As well, epilepsy diagnosis, lines of treatment, importance of adherence to treatment, dose and side effects of antiepileptic drugs.

#### The second session

focused on informing mothers about seizure management before, during, and after the attack.

#### The third session

A practical session that focus on empowering mothers’ demonstration of first aid of epilepsy before, during, and after seizure.

#### The fourth session

The aim of this session is to help mothers to eliminate the care burden of children’s care through reducing the physical, emotional, psychological, and social stress associated with caregiving responsibilities. Mothers were allowed to discuss their burden, empowering mothers through education, skill, emotional support to feel more confident and supported.

### Evaluation phase

Mothers’ knowledge, reported practice, and care burden immediately post implementation of FCEM, and after three months (post-test), then post-test were compared to pre-test (baseline data).

### Statistical analysis

Organization, tabulation and statistical analysis of the collected date were done using SPSS software (Statistical package for the Social Sciences version 26, SPSS Inc. Chicago, II, USA, 2019). The range, mean and standard deviation were calculated for quantitative data. Evaluation of the correlation between variables was done using Pearson’s correlation coefficient (r). Statistical significance was adopted at *P* < 0.05 (*), highly significance was adopted at *P* < 0.001(**).

## Results

Table 1 presents mothers’ socio-demographic characteristics. It was observed that, mean ± SD of mothers’ age was 33.616 ± 8.22 years, 38.3% of the participated mothers had secondary education. Nearly two thirds of the mothers (61.7%, 63.3%) lived in rural areas, and didn’t work respectively. The majority of the mothers (96.7%) had low-income.

Table 2 demonstrates children’ socio-demographic characteristics. The mean ± SD of children’ age was 6.150 ± 1.109. It was found that 61.7% were males & more than half of them (51.7%) were the first child for their parents.

Total knowledge scores of the mothers about epilepsy and epileptic seizures were displayed in Table 3. More than one-third of the mothers (36.7%) had high knowledge score about epilepsy and epileptic seizures before implementing FCEM compared to all of the studied mothers (100%), and 95% of them immediately and three months after implementing FCEM respectively.

Table 4 portrayed total mothers’ reported practice regarding epilepsy and epileptic seizures before, immediately post, and three months after FCEM implementation. It was clear that, only few percentages of the studied mothers (1.7%) had satisfactory practice before implementing FCEM which improved to 91.7%, and 100% immediately after FCEM implementation and three later respectively.

Table 5 explained total mothers’ reported care burden regarding epilepsy before, immediately post and three months post FCEM. It was apparent that, 66.7% had severe burden which remarkably decreased to 16.6% immediately post implementing FCEM and none of the mothers three months after implementing FCEM was experienced severe burden.

**Table 1:** Percentage distribution of the mothers’ socio- characteristics. (*n* = 60)

Socio-demographic characteristics	Mothers (*n* = 60)	
	no.	%
**Age (years)**		
Less than 20	1	1.7
20<30	19	31.7
30<40	24	40.0
≥ 40	16	26.6
**Mean ± SD**	**33.616 ± 8.22**	
**Level of education**		
Illiterate	5	8.3
Primary school	16	26.7
Secondary or technical school	23	38.3
University school	16	26.7
**Residence**		
Rural	37	61.7
Urban	23	38.3
**Job**		
Not working	38	63.3
Working	22	36.7
**Income**		
Low level	58	96.7
Moderate level	2	3.3

**Table 2:** Percentage distribution of children’s socio-demographic characteristics. (*n* = 60)

Children’s socio-demographic characteristics	Children(*n* = 60)	
	no.	%
**Age (years)**		
4<6	21	35.0
6–8	39	65.0
**Mean ± SD**	**6.150 ± 1.109**	
**Childs’ sex**		
Male	37	61.7
Female	23	38.3

**Table 3:** Total knowledge scores of the mothers about epilepsy and epileptic seizures. (*n* = 60)

Mothers’ total knowledge level	Before FCEM		Immediately post FCEM		Three months after FCEM		χ^2^ *P*_1_	χ^2^ *P*_2_	χ^2^ *P*_3_
	no.	%	no.	%	no.	%			
Low level Moderate level High level	5 33 22	8.3 55.0 36.7	0 0 60	0.0 0.0 100.0	0 3 57	0.0 5.0 95.0	55.610 0.0001**	45.506 0.0001**	3.077 0.079
Mean ± SD	13.033 ± 2.79		19.216 ± 1.35		19.033 ± 1.71		F value = 177.148 ***P*** = 0.0001**		

**χ**^**2**^ **=** Chi-square test

* Statistically significant at (*P* < 0.05)

** Highly statistically significant at (*P* < 0.001)

P_1_: before and immediately post, P_2_: before and three months post, P_3_: immediately and three months post

**Table 4:** Total mothers’ reported practice scores regarding epilepsy and epileptic seizures. (*n* = 60)

Mothers’ total reported practice	Before FCEM		Immediately after FCEM		After three months FCEM		χ^2^ *P*_1_	χ^2^ *P*_2_	χ^2^ *P*_3_
	No	%	No	%	No	%			
Unsatisfactory practice Satisfactory practice	59 1	98.3 1.7	5 55	8.3 91.7	0 60	0.0 100.0	97.634 **0.0001** **	116.066 **0.0001** **	5.217 0.022*
Mean ± SD	15.566 ± 4.763		31.300 ± 2.789		33.016 ± 1.478		**F value = 509.951** ***P*** = 0.0001**		

**χ**^**2**^ **=** Chi-square test

**F value** = one way ANOVA test

*Statistically significant at (*P* < 0.05)

** Highly statistically significant at (*P* < 0.001)

P_1_: before and immediately post, P_2_: before and three months post, P_3_: immediately and three months post

**Table 5:** Mothers’ reported total levels of care burden regarding epilepsy. (*n* = 60)

Mothers’ total levels of reported care burden	Before FCEM		Immediately after FCEM		Three months after FCEM		χ^2^ *P*_1_	χ^2^ *P*_2_	χ^2^ *P*_3_
	No	%	No	%	No	%			
Mild burden.	0	0.0	1	1.7	55	91.7	**31.188**	**104.00**	97.923
Moderate burden	20	33.3	49	81.7	5	8.3	0.0001**	0.0001**	0.0001**
Severe burden	40	66.7	10	16.6	0	0.0			
Mean ± SD	62.316 ± 5.682		54.066 ± 6.294		36.483 ± 4.692		**F value = 333.641** ***P*** = 0.0001**		

**χ**^**2**^ **=** Chi-square test

**F value** = one way ANOVA test

*Statistically significant at (*P* < 0.05)

** Highly statistically significant at (*P* < 0.001)

P_1_: before and immediately post, P_2_: before and three months post, P_3_: immediately and three months post

## Discussion

Epilepsy is one of the commonest neurological disorders. It is caused by spontaneous repeated seizures resulted from increased brain electrical activity. It occurs when a child has 2 or more seizures with unknown cause [4]. & [25] Epilepsy is a neurological disorder that leads to cognitive, psychological, in addition to social complications. It is an emergent and life-threatening condition due to its high rate of illness and mortality [30]. 

Taking care of an epileptic children forms a heavy task among family members, especially mothers. Mothers may suffer from anxiety, frustration or depression as they struggle to exert greater effort to care for their epileptic children. The burden among mothers may also increase due to their lower level of education, incompetent care of their epileptic children, and chronicity of the disease. The emotional suffering, they endure impacted their care quality which in turn can have an impact on the children`s prognosis. [18] So, this study intended to evaluate the effect of FCEM on the care burden between mothers looking after their epileptic children.

It was noticed that, greater than one-third of the mothers were highly knowledgeable about epilepsy and epileptic seizures before implementing FCEM. This may be due to low education of the mothers, whereas more than one quarter of them had primary education. Also, about two-thirds of the mothers resided in rural areas, and nearly all of them had low income level. All these factors may precipitate low knowledge level.

This result was congruent with Turan et al., (2022) [31] in their conducted study about ″effect of educational interventions on epilepsy knowledge in epileptic children and their parent″, and another study conducted by Akbas et al., (2022) [32] entitled ″ knowledge, attitude, and parent’s behaviors regarding epilepsy″ as they proved that, the parents in their studies had poor knowledge regarding epilepsy.

This study showed that, mothers’ total knowledge about epilepsy and epileptic seizures was enhanced immediately and three months after implementing FCEM. The positive effect of FCEM program which based on mothers’ needs, good preparation of mothers, and mothers’ enthusiasm to be more knowledgeable about epilepsy could be the causes.

Several studies conducted by Turan et al., (2022), Akbas et al., (2022) [31, 32] were in harmony with such findings. They reported enhancement of parents’ knowledge after the program. These findings were consistent with Nagan et al., (2017) [33] who conducted a study to ″evaluate parental knowledge and misconceptions about epilepsy’’ and reported that, parents’ knowledge increased after the intervention. Also, Hagemann et al., (2022) [34] whose study evaluate the ″efficacy of an educational program for parents of epileptic children’’ was in agreement with the current findings, as they stated that, parents knowledge was enhanced after educational program.

Concerning mothers’ total reported practice regarding epilepsy and epileptic seizures. The majority of the participated mothers in this study their practice was unsatisfactory before implementing FCEM. These findings could be explained by the association between knowledge and practice as only higher than one-third of the mothers were high in their knowledge about epilepsy and epileptic seizures which in turn affected their practice negatively. In addition to, nearly three quarters of the mothers weren’t educated or had primary education or secondary education that consequently affected their caring practice for their epileptic children as All of these factors may be significant causes of low practice scores before implementing FCEM.

These present results were in harmony with Shasha et al., (2022) [35] who studied ″ effect of simulation training on seizure management and level of anxiety between mothers of epileptic children″ reported that, mothers’ management of their children’s seizures were incompetent before the training program. Another study conducted by Shahin et al., (2021) [36] who evaluated″ effect of a structured educational intervention on knowledge, attitude, practice, and self-efficacy of caregivers of epileptic children″ were congruent with these current findings. They found that, caregivers’ practices were low in seizures management before implementing the program. On contrary, Badawy et al., (2022) [37] who implemented a study to assess ″maternal care of their epileptic children″ stated that, total practice of half of their studied mothers were good.

On the other hand, significant enhancement in mothers reported practice related to epilepsy and epileptic seizures immediately and three months after FCEM implementation was found. This may be explained on the basis of the improvement of the knowledge of mothers, and efficiency of FCEM in empowering mothers with needed skills to be able to manage their epileptic children. In accordance with this finding was Sheijani et al., (2020) [20] whose study about ″ effect of creating empowerment opportunities for parents of epileptic children″ ruled out that, parents’ management of seizure attacks was enhanced after the empowerment program. Another studies performed by Shasha et al., (2022), Shahin et al., (2021) [35, 36] were matched with the present results. They found that, caregivers’ practices about seizures management were increased after implementing the educational program.

Regarding mothers’ total reported care burden regarding epilepsy. This study found that, about two-thirds of the mothers had severe burden before implementing FCEM. This finding was attributed to mothers’ poor knowledge, and unsatisfactory practice regarding epilepsy and seizures before implementation of FCCM were consequently increased care burden level as mothers didn’t have the skill or knowledge to manage the problems facing their children that leads to increase care burden.

This finding was in compliance with Balouchi et al., (2021) [38] who studied ″effect of psycho-educational program on caregivers burden of epileptic children″. They stated that, families with children diagnosed with epilepsy had significantly high level of care burden before the intervention. Also, Ozer, (2021) [39] who evaluated ″care burden in caregivers of epileptic patients ″ declared that, caregivers suffered from moderate to severe care burden before the intervention.

On contrary, Pokharel, (2020) [40] who performed a study to assess ″ burden and its associated predictors between caregivers of epileptic patients″ opposed this current finding. They found that, greater than half of the studied sample had no to mild care burden. The differences in burden level among the previous studies could be a due to cultural and socioeconomic disparities, the accessibility of therapeutic alternatives, children age, and presence of support either from family members or from the community.

On the other hand, there was remarkable decrease in mothers’ reported care burden scores regarding epilepsy and epileptic seizures immediately and three months after FCEM implementation. This may be explained as increased mothers’ knowledge led to improve their self-confidence, drive, and caregiving abilities while reducing stress. As well, decrease mothers’ feeling of inadequacy and embarrassment which in turn decreased mothers’ reported care burden.

A study implemented by Barakat et al., (2024) [41] to evaluate ″effect of psychological empowerment program on burden and self-efficacy of mothers of epileptic children″ supported the current finding. As they mentioned that, there was an important decrease in care burden levels of the mothers post implementation of the empowerment program. In addition to, Gu, (2023) [23] who studied ″parents’ experiences of caring children with epilepsy″ agreed with such finding. They revealed that, total care burden of the studied sample decreased post implementation of the intervention.

## Conclusion

According to the results of this present study, it may be stated that, Mothers’ knowledge and reported practice had been improved after implementing FCCM compered to pre- implementation of the model. There was a significant decrease in the care burden experienced by mothers after implementing FCCM for their children with epilepsy immediately after implementing FCEM, and post three months as compared to pre implementing the model.

### Recommendations

According to the findings of this study, the following suggestions are presented:

- Continuous educational programs based on Family- Centered Empowerment Model should be conducted to enhance mothers’ knowledge about epilepsy and seizure management.
- Simple and culturally appropriate educational materials-Booklet and brochures in Arabic- should be made available in Neurology Unit at the Pediatric Medical Department to support parents’ learning.
- Integrate FCEM into routine nursing practice to improve quality of care for children with epilepsy.
- Provide counseling services and psychological support including comprehensive psychological services, stress management support, should be offered to assist mothers in coping with caregiving burden, anxiety and fatigue.
- Training family members in epilepsy care to promote caregiving responsibilities therby reducing physical and emotional burden on mothers.
- A collaborative multi-disciplinary approach should be adopted to foster trust, improve communication with families, and ensure continuity and coordination of care.

## Data Availability

No datasets were generated or analysed during the current study.
